# Tracking natural and anthropogenic Pb exposure to its geological source

**DOI:** 10.1038/s41598-018-20397-y

**Published:** 2018-01-31

**Authors:** Jane Evans, Vanessa Pashley, Richard Madgwick, Samantha Neil, Carolyn Chenery

**Affiliations:** 1 NERC Isotope Geosciences Laboratory, British Geological Survey, Keyworth, Nottingham, UK NG12 5GG; 2 Department of Archaeology, Cardiff University, Cardiff, Wales CF10 3AT UK; 3 Department of Archaeology, Durham University, Durham, DH1 3LE England

## Abstract

Human Pb exposure comes from two sources: (i) natural uptake through ingestion of soils and typified by populations that predate mining activity and (ii) anthropogenic exposure caused by the exposure to Pb derived from ore deposits. Currently, the measured concentration of Pb within a sample is used to discriminate between these two exposure routes, with the upper limit for natural exposure in skeletal studies given as 0.5 or 0.7 mg/kg in enamel and 0.5/0.7 μg/dL in blood. This threshold approach to categorising Pb exposure does not distinguish between the geological origins of the exposure types. However, Pb isotopes potentially provide a more definitive means of discriminating between sources. Whereas Pb from soil displays a crustal average ^238^U/^204^Pb (μ) value of c 9.7, Pb from ore displays a much wider range of evolution pathways. These characteristics are transferred into tooth enamel, making it possible to characterize human Pb exposure in terms of the primary source of ingested Pb and to relate mining activity to geotectonic domains. We surmise that this ability to discriminate between silicate and sulphide Pb exposure will lead to a better understanding of the evolution of early human mining activity and development of exposure models through the Anthropocene.

## Introduction

Lead (Pb) is a poisonous element that causes diseases of the nervous, digestive and reproductive systems. Humans are exposed to Pb through natural and anthropogenic routes^1^. Natural exposure to Pb, exemplified by populations that predate ore extraction is, typically, through accidental hand to mouth ingestion of soil, particularly during childhood^2^. Anthropogenic exposure predominantly arises as a result of interaction with Pb released into the environment through the mining and use of Pb-bearing sulphide deposits. This latter exposure route is diverse and historically includes such things as (i) the use of Pb water pipes, (ii) the use of Pb as a sweetener in food and drink, (iii) Pb added to paint and makeup, and, more recently, (iv) Pb additives in petrol^3,4^. Exposure to anthropogenic Pb typically results in elevated blood (and thus tooth) Pb levels^5^. It is this elevated Pb concentration that is currently used to differentiate between individuals exposed to anthropogenic versus natural Pb sources. The upper limit for natural exposure in skeletal studies given as 0.5^5^ or 0.7 mg/kg^4,6^ in enamel (equating to 0.5/0.7 μg/dL in blood)^7^. However, truly understanding the uptake pathways is important for prevention of exposure and, in archaeological studies, understanding human cultural development. This paper demonstrates that the different geological processes (see appendix) that control the Pb isotope composition of the silicate (natural) and sulphide (anthropogenic) Pb sources provide a more precise way of distinguishing between the two pathways.

The basic principles, and method of data display for the U-Pb isotope systems, can be found in a number of texts^8–10^. The parent isotopes ^235^U, ^238^U and ^232^Th decay over geological time to produce the daughter products ^207^Pb, ^206^Pb and ^208^Pb, respectively, with one stable isotope of Pb, ^204^Pb, used as the invariant reference isotope. The traditional method of data display, utilized in many archaeological studies, is to plot the ^207^Pb/^206^Pb and ^208^Pb/^206^Pb ratios and describe compositional fields within this bivariate space. However, it has been noted that this method of representation tends to compress the data and make it a relatively poor discriminant because conformable Pb ore deposits have a very restricted range on this type of plot the inclusion of the ^204^Pb ratios helps multiple source mixing to be identified^11^. While the data from this paper can be seen plotted in this conventional manner in the appendix, we have preferred to employ a recently suggested graphical method of displaying the time-integrated ^238^U/^204^Pb (μ) as a function of the Pb model age (*T*). A full description of this method and the equations needed to undertake the calculations are given in Albarede *et al*. (2012)^12^. This method requires the derivation of the two axis variables from the measured Pb isotope compositions and is therefore more complex that the conventional ^207^Pb/^206^Pb and ^208^Pb/^206^Pb representation. However, it has the distinct advantage in that it provides information about the geological origins of the sample without recourse to reference datasets. Ore forming events are generally related to major geological mountain building processes of which three dominate European geology^13^; the Alpine event of c. 60–2.5 Ma which is most evident in circum-Mediterranean geology; the Hercynian c. 280–380 Ma, which mostly affects northern continental Europe and southern Britain and the Caledonian event of c. 390–490 Ma seen in the Palaeozoic and older rocks of Britain and Scandinavia. The calculation of Pb model age gives an estimate of the age and hence geological episode to which mineralisation is associated. Mu (μ) provides evidence of the geochemical nature of the source rock of the mineralization. For example, deposits such as those in Tunisia^14^, source their Pb from uranium rich granite domains and hence have elevated ^238^U/^204^Pb (μ) values, whereas Pb derived from more basic/ultrabasic deposits, such as are found in Cyprus, reflect the low uranium nature of the host with low ^238^U/^204^Pb (μ) values^14^. The combination of the model age (*T*) and ^238^U/^204^Pb (μ) thus provide geological, and hence geographic, constraints on the origin of the Pb without recourse to large reference datasets.

In this study, the transfer of labile soil Pb into fauna is primarily demonstrated using Neolithic (pre-anthropogenic Pb) pigs teeth. Pigs ingest soil while grubbing for food and hence provide a simple transfer model. The animals are from the Neolithic feasting site of Durrington Walls in southern England. These data are supplemented by two human ‘natural exposure’ populations: (i) a dataset of Neolithic individuals from British archaeological sites, and (ii) 10^th^ century individuals, all typified by very low Pb concentration levels (0.11 ± 0.18 mg/kg, 2 SD, n = 34)^15,16^. These are then compared with data from three Early (5–7^th^ century) Anglo Saxon and Anglian sites in England, where elevated Pb concentrations are suggestive of anthropogenic Pb exposure. The sites, and the average Pb concentrations in the tooth enamel, are as follows: Berinsfield^17^ in central England, where individuals have average tooth enamel Pb concentrations of 2.5 ± 4 mg/kg (2 SD, n = 11); Eastbourne in southern England, where individuals have average tooth enamel Pb concentrations of 6 mg/kg ± 22 mg/kg (2 SD, n = 21) and West Heslerton, north eastern England, which straddles the natural/anthropogenic Pb exposure boundary (0.7 ± 2.8 mg/kg; 2 SD, n = 33)^18^.

## Method Section

Tooth enamel samples were prepared as follows: The enamel surface of the tooth was abraded from the surface to a depth of >100 microns using a diamond coated dental bur and the removed material discarded. An enamel sample was cut from the tooth using a flexible diamond edged rotary dental saw. All surfaces were mechanically cleaned with a diamond bur to remove adhering dentine. The resulting sample was transferred to a clean (class 100, laminar flow) working area for further preparation. In a clean laboratory, the sample was cleaned ultrasonically in high purity water to remove dust. It was then rinsed twice in de-ionized water, and soaked for an hour at 60 °C, before rinsing again and then leaching for 5 minutes with Teflon distilled 0.2 M HCl,. After a final rinse, the sample was dried and transferred into a pre-cleaned Teflon beaker where it was dissoslved in Teflon distilled 8MHNO3, evaporated to dryness and converted to bromide form using Romil© UpA HBr. Soils were leached with deionised water for 24 hours, centrifuged and the supernatant decanted into clean Savillex Beakers, evaporated to dryness and converted to bromide form as before. Separation of Pb from samples was undertaken using standard ion exchange techniques. The data in this paper has been acquired over a number of years and includes lead isotope compositions that were determined by either thermal ionisation mass spectrometry (TIMS) using a Finnigan Mat 262, or multi-collector inductively coupled plasma mass spectrometry (MC-ICP-MS) using a Nu Plasma HR or a Thermo Fisher Scientific Neptune Plus. TIMS Pb was run using rhenium filaments in a silica gel-phosphoric acid. Lead blanks were c 70 pg. Lead isotope ratios were normalised to values of NBS 981^19^, which gave the following reproducibility during the period of analysis: ^206^Pb/^204^Pb = 0.20%, ^207^Pb/^204^Pb = 0.29%, ^208^Pb/^204^Pb = 0.40% (2σ, n = 31). Samples analysed by MC-ICP-MS were spiked with a thallium (Tl) solution and introduced into the instrument via an ESI 50 μl/min PFA micro-concentric nebuliser attached to a de-solvating unit (Nu Instruments DSN 100 or Cetac Aridus II) and normalised to NBS981^20^. Average 2 SD reproducibility for the following ratios is ^206^Pb/^204^Pb = 0.008%; ^207^Pb/^204^Pb = 0.008%; ^208^Pb/^204^Pb = 0.009%. The pig enamel samples, which were analytically challenging due to low Pb yields, were run on the Neptune using a high sensitivity jet cone and reproducibility was ^206^Pb/^204^Pb = 0.027% %; ^207^Pb/^204^Pb = 0.031%; ^208^Pb/^204^Pb = 0.041%. Lead concentrations, where documented, were measured by either isotope dilution^8^ or solution plasma^21^. Details of the samples and sites and extended methodology are supplied in the supplementary information section. Data is presented in Table 1.

**Table 1:** The primary Pb isotope ratios of all samples discussed in this study are presented in the table.

Sample	^206^Pb/^204^Pb	^207^Pb/^204^Pb	^208^Pb/^204^Pb	*T (Ma)*	^238^U/^204^Pb (μ)	Pb ppm
modern soil leaches						
BF-S1 (HCl)*	18.47	15.63	38.44	209	9.71	nd
BF-S1 (acetic)*	18.46	15.63	38.43	222	9.72	nd
GEMAS 003	18.3886	15.6506	38.4359	313	9.82	nd
GEMAS 005	19.1357	15.7032	38.8143	−140	9.88	nd
GEMAS 006	18.5291	15.6469	38.4257	207	9.78	nd
GEMAS 012	18.5943	15.6516	38.5632	161	9.77	nd
GEMAS 014	18.2826	15.6144	38.2266	322	9.70	nd
GEMAS 033	19.0200	15.6827	38.4305	−96	9.81	nd
GEMAS 040	18.1044	15.6075	38.0741	441	9.71	nd
GEMAS 044	18.7172	15.6675	38.7582	100	9.81	nd
GEMAS 057	18.5600	15.6476	38.3810	178	9.76	nd
GEMAS 062	18.7401	15.6705	38.7513	89	9.82	nd
GEMAS 063	18.5443	15.5970	38.1120	90	9.57	nd
GEMAS 074	18.3499	15.6584	38.4808	354	9.85	nd
GEMAS 072	18.0606	15.6065	38.0179	472	9.72	nd
GEMAS 108	18.2325	15.6467	38.2883	419	9.84	nd
GEMAS 118	18.9767	15.6861	38.7838	−57	9.83	nd
MW-C (acetic)*	18.96	15.66	38.78	−102	9.73	nd
MW-C*	18.92	15.66	38.70	−71	9.74	nd
MW-S1 (HCl)*	18.98	15.66	38.81	−113	9.74	nd
MW-S1 (acetic)*	18.99	15.68	38.77	−84	9.80	nd
MW-S2 (HCl)*	19.02	15.66	38.84	−156	9.71	nd
MW-S2 (acetic)*	19.01	15.67	38.76	−107	9.78	nd
SK2-S*	18.46	15.60	38.37	166	9.62	nd
SK2-S (acetic)*	18.47	15.62	38.39	190	9.67	nd
WH-C (acetic)*	18.62	15.60	38.62	35	9.57	nd
WH-S (acetic)*	18.17	15.57	38.03	332	9.56	nd
WH-S*	18.32	15.59	38.21	237	9.57	nd
WIN-C (HCl)*	18.82	15.64	38.60	−44	9.67	nd
Ancient soils						
F23A (72)*	18.8828	15.6555	38.6290	−40	9.75	nd
F23A (73)*	18.7806	15.6420	38.5729	−12	9.68	nd
F23B (74)*	18.8766	15.6473	38.6283	−48	9.73	nd
F23B (75)*	18.8195	15.6275	38.5960	−48	9.66	nd
F23C (76)*	18.6285	15.6262	38.4747	85	9.67	nd
F23D (77)*	18.9089	15.6413	38.6393	−98	9.68	nd
F23E (78)*	18.8305	15.6320	38.6031	−57	9.65	nd
Mangots field SK1a*	18.44	15.64	38.44	258	9.77	nd
Mangots field SK1b*	18.43	15.63	38.39	239	9.71	nd
Mangots field SK2a*	18.41	15.62	38.36	227	9.67	nd
Mangots field SK2b*	18.42	15.64	38.41	257	9.75	nd
S0025	18.47	15.64	38.46	235	9.76	nd
BH 2551d*	18.40	15.63	38.38	267	9.74	nd
blackfriars 209*	18.41	15.62	38.36	225	9.67	nd
Blackfriars 341 (4)*	18.50	15.66	38.50	242	9.81	nd
Blackfriars 341 (5)*	18.46	15.63	38.44	219	9.72	nd
Blackfriars 357*	18.41	15.62	38.38	236	9.69	nd
Blackfriars 77*	18.44	15.63	38.40	235	9.72	nd
FGH 045d*	18.46	15.62	38.41	208	9.69	nd
FGH 218d*	18.47	15.62	38.45	201	9.69	nd
Eagle Hall G318*	18.42	15.62	38.36	218	9.67	nd
Eagle Hall G319*	18.42	15.62	38.36	219	9.67	nd
Eagle Hall G326*	18.41	15.61	38.35	218	9.65	nd
Eagle Hall G339 (65)*	18.43	15.62	38.40	230	9.70	nd
Eagle Hall G339 (66)*	18.42	15.61	38.40	217	9.66	nd
wasp 190d	18.46	15.63	38.42	227	9.73	nd
wasp 42d	18.44	15.63	38.40	231	9.71	nd
BIP-SK109d	18.43	15.62	38.41	225	9.69	nd
BIP-SK164d	18.57	15.64	38.45	150	9.72	nd
BIP-SK198d	17.74	15.57	37.58	653	9.69	nd
BIP-SK199d	18.57	15.64	38.37	155	9.73	nd
BIP-SK259d	18.46	15.65	38.48	253	9.78	nd
BIP-SK269d	18.09	15.62	37.92	481	9.78	nd
BIP-SK212d	17.50	15.54	37.18	769	9.62	nd
Neolithic pig enamel						
DWP04A	18.0875	15.6150	38.0288	472	9.76	nd
DWP05A	18.2236	15.6237	38.1570	384	9.75	nd
DWP07A	18.2780	15.6247	38.1706	344	9.74	nd
DWP13A	18.4207	15.6338	38.3297	256	9.74	nd
DWP15A	18.2120	15.6295	38.1987	402	9.77	nd
DWP22A	18.3961	15.6312	38.3168	269.7	9.737374	nd
DWP24A	18.2378	15.6443	38.1944	410.4	9.826681	nd
DWP26A	18.3790	15.6437	38.3587	305.3	9.789326	nd
DWP27A	18.3942	15.6466	38.3177	299.6	9.797292	nd
DWP32A	18.2833	15.6363	38.2268	362.2	9.782787	nd
DWP35A	18.3465	15.6435	38.3721	329	9.796234	nd
DWP36A	18.2885	15.6378	38.2846	361	9.787372	nd
DWP37A	18.2465	15.6355	38.2055	387.8	9.788775	nd
DWP39A	18.4926	15.6506	38.4216	234.1	9.790358	nd
DWP45A	18.1385	15.6223	38.0950	443.2	9.763928	nd
DWP46A	18.4686	15.6467	38.3827	244.5	9.780426	nd
DWP54A	18.1633	15.6323	38.1186	443.3	9.797941	nd
DWP55A	18.1463	15.6306	38.1413	452.8	9.795715	nd
DWP62A2	18.2598	15.6350	38.2061	377.2	9.783656	nd
DWP69A2	18.5259	15.6503	38.4690	208.9	9.782169	nd
DWP71A2	18.3868	15.6247	38.2877	263.3	9.71193	nd
Neolithic human enamel						
WHIT534-LM2	18.2387	15.6160	38.1814	357	9.71	nd
WHIT512/2-LM2	18.2635	15.6128	38.1553	333	9.69	nd
WHIT487-RM2	18.4413	15.6257	38.3583	225	9.70	nd
WHIT451-RM1	18.2717	15.6190	38.1454	338	9.72	nd
WHIT957-LM1	18.3921	15.6061	38.3127	223	9.64	nd
WHIT957-RM3	18.4434	15.6260	38.3510	224	9.70	nd
39.190/148b (LM3)	18.1207	15.6149	38.0092	443	9.74	nd
39.190/148b (LM2)	18.2493	15.6284	38.2022	373	9.76	nd
39.190/201 (LM3)	18.2430	15.6244	38.1816	370	9.75	nd
39.190/201 (LM2)	18.2947	15.6253	38.2427	333	9.74	nd
Low ppm						
GRIS**	18.4562	15.6340	38.4396	229	9.73	0.003
AB 61231 M3	18.7064	15.6460	38.5129	65	9.73	na
AB 61231 P2	18.7824	15.6539	38.6014	24	9.75	na
MH05 1861**	18.32	15.62	38.31	304	9.71	0.25
MN04–897**	18.35	15.63	38.42	301	9.74	0.31
WEY08 SK3694	18.5035	15.6368	38.4389	199	9.73	0.25
WEY08 SK3696	18.6622	15.6504	38.5264	107	9.75	0.20
WEY08 SK3704	18.6646	15.6459	38.5414	97	9.74	0.11
WEY08 SK3705	18.6319	15.6439	38.5044	117	9.74	0.02
WEY08 SK3706	18.6062	15.6376	38.5048	124	9.72	0.17
WEY08 SK3707	18.7242	15.6657	38.6022	91	9.80	0.13
WEY08 SK3710	18.6161	15.6405	38.4681	122	9.73	0.09
WEY08 SK3711	18.6048	15.6414	38.4444	133	9.73	0.11
WEY08 SK3712	18.50784	15.6415	38.1849	47	9.74	0.08
WEY08 SK3720	18.7399	15.6493	38.5764	47	9.74	0.09
WEY08 SK3722	18.7072	15.6516	38.5298	76	9.75	0.08
WEY08 SK3724	18.7826	15.6485	38.5909	13	9.73	0.15
WEY08 SK3725	18.4175	15.6294	38.3687	250	9.72	0.03
WEY08 SK3726	18.4979	15.6363	38.4385	203	9.73	0.22
WEY08 SK3730	18.4881	15.6266	38.4501	191	9.70	0.26
WEY08 SK3733	18.8172	15.6589	38.6805	8	9.76	0.05
WEY08 SK3738	18.6203	15.6488	38.4946	136	9.76	0.03
WEY08 SK3739	18.5716	15.6366	38.4751	148	9.72	0.36
WEY08 SK3743	18.4491	15.6317	38.3637	230	9.73	0.14
WEY08 SK3744	18.6249	15.6391	38.4832	113	9.72	0.10
WEY08 SK3746	18.6515	15.6480	38.5372	111	9.75	0.04
WEY08 SK3747	18.8180	15.6578	38.6595	5	9.75	0.05
WEY08 SK3749	19.1044	15.6846	38.8125	−156	9.81	0.05
WEY08 SK3751	18.5257	15.6383	38.4672	186	9.74	0.16
WEY08 SK3752	18.3704	15.6213	38.2301	269	9.70	0.03
WEY08 SK3757	18.1792	15.6213	38.0693	411	9.75	0.09
WEY08 SK3758	18.7095	15.6547	38.6437	80	9.76	0.11
WEY08 SK3759	17.5988	15.5684	37.4141	746	9.71	0.05
WEY08 SK3760	18.5324	15.6395	38.4054	183	9.74	0.03
WEY08 SK3761	18.5092	15.6408	38.4393	203	9.75	0.02
ASDR-ADV-1^†^	18.0443	15.6290	37.8912	524	9.75	0.04
HUMAN ANTHROPOGENIC EXPOSURE						
Berinsfield						
Ber 010i	18.40	15.52	38.07	36	9.29	0.33
Ber 026	18.36	15.52	38.04	69	9.30	1.44
Ber 020	18.37	15.52	38.06	71	9.31	1.41
Ber 006	18.36	15.52	38.06	81	9.32	1.00
Ber 005	18.40	15.53	38.11	68	9.34	0.44
Ber 042	18.35	15.54	38.08	122	9.39	3.14
Ber 054	18.37	15.54	38.11	106	9.39	6.94
Ber 004	18.37	15.54	38.11	109	9.39	5.14
Ber 061	18.42	15.55	38.14	97	9.42	0.53
Ber 018	18.41	15.55	38.20	109	9.43	1.83
Ber 081	18.37	15.55	38.15	134	9.44	5.54
Ber 152	18.39	15.56	38.17	132	9.45	2.44
Ber 030	18.40	15.56	38.17	131	9.47	3.27
Ber 073	18.39	15.57	38.19	146	9.49	2.74
Ber 008	18.40	15.57	38.19	141	9.49	5.69
Ber 049	18.42	15.57	38.21	137	9.51	1.88
Ber 150/1	18.43	15.60	38.32	185	9.61	0.81
Ber 001	18.45	15.60	38.34	176	9.62	1.16
Ber 141/1	18.44	15.64	38.43	246	9.75	2.59
Eastbourne						
EAS-796	18.28	15.59	38.15	273	9.59	0.90
EAS-270	18.44	15.60	38.30	182	9.61	4.70
EAS-264	18.37	15.60	38.33	225	9.61	49.98
EAS-57	18.47	15.62	38.42	192	9.68	1.84
EAS-753	18.45	15.62	38.38	210	9.68	0.87
EAS-67	18.47	15.62	38.40	197	9.68	3.76
HR2/1992-2 (HF-01)	18.40	15.62	38.36	246	9.69	8.60
ECE97b 777 (HF-02)	18.41	15.62	38.37	243	9.70	7.90
EAS-157	18.54	15.63	38.47	169	9.72	0.22
EAS-233	18.43	15.63	38.47	241	9.72	0.80
EAS-111	18.43	15.63	38.43	247	9.74	2.70
BH 1959 (HF-05)	18.38	15.63	38.38	285	9.75	5.50
EAS-64	18.48	15.64	38.46	224	9.76	9.38
EAS-190	18.53	15.66	38.53	221	9.81	0.48
EAS-61	18.50	15.66	38.47	244	9.81	0.24
EAS-355	18.52	15.71	38.43	321	10.01	0.28
EAS-309	18.46	15.72	38.34	377	10.06	17.66
EAS-650	18.46	15.74	38.42	417	10.15	0.99
EAS-381	18.45	15.76	38.34	471	10.25	0.77
EAS-681	18.46	15.78	38.41	498	10.33	2.70
EAS-481	18.47	15.80	38.42	515	10.38	13.24
West Heslerton*						
G73	18.53	15.61	38.43	135	09.6339	0.19
G74	18.44	15.59	38.32	168	09.5821	0.37
G75	18.45	15.60	38.32	167	09.5974	8.16
G78	18.46	15.62	38.37	192	09.6621	1.66
G84	18.47	15.63	38.39	219	09.7258	0.20
G89	18.38	15.59	38.25	190	9.5600	0.21
G97	18.38	15.61	38.30	246	09.6627	0.13
G97	18.41	15.58	38.30	161	09.5432	0.19
G98	18.24	15.60	38.12	321	09.6441	0.49
G100	18.44	15.60	38.34	182	9.619521	0.41
G102	18.48	15.64	38.44	231	9.770345	0.21
G101	18.47	15.63	38.40	210	9.71591	0.48
G109	18.49	15.61	38.40	155	9.621422	0.26
G113	18.43	15.59	38.29	159	9.557591	0.23
G114	18.51	15.62	38.41	156	9.648991	0.26
G115	18.43	15.60	38.33	182	9.609438	0.99
G117	18.48	15.61	38.40	164	9.630817	0.37
G122	18.42	15.59	38.28	168	9.564897	0.87
G132	18.44	15.59	38.30	146	9.548203	0.26
G133	18.43	15.59	38.28	158	9.554169	2.96
G139	18.50	15.63	38.40	185	9.695241	0.15
G144	18.50	15.61	38.41	159	9.64807	0.28
G145	18.47	15.59	38.33	135	9.557071	0.14
G149	18.47	15.60	38.34	149	9.587468	0.55
G151	18.58	15.61	38.49	83	9.60852	0.28
G154	18.45	15.61	38.36	190	9.641601	0.26
G158	18.43	15.62	38.40	224	9.684619	0.49
G159	18.51	15.62	38.41	159	9.656622	0.15
G162	18.44	15.61	38.36	186	9.630184	0.94
G164	18.52	15.64	38.51	190	9.742234	0.41
G166	18.42	15.59	38.32	176	9.574549	0.35
G169	18.49	15.64	38.46	219	9.752405	0.19
G173	18.42	15.61	38.32	197	9.627136	0.21

Data produced by TIMS analysis is quoted to two decimal places and data derived from plasma analysis is quoted to four decimal places. Previously published data are indicated as follows: *Montgomery 2002^18^, ^†^Harris *et al*. 2017^31^, **Montgomery *et al*. 2011^4^. Pb concentrations are given, where available. The majority of soil leaches are water based but where dilute HCl or Acetic acid were used this is indicated. The ancient soil compositions are based on analyse of dentine which re-equilibrates with its environment during burial. Model age (T_DM_), and ^238^U/^204^Pb (μ), values are calculated for all samples using the method described in Albarede *et al*. 2012^12^. The iterations involved in the calculation were completed at better that x*E^−6^.

**Figure 1:**
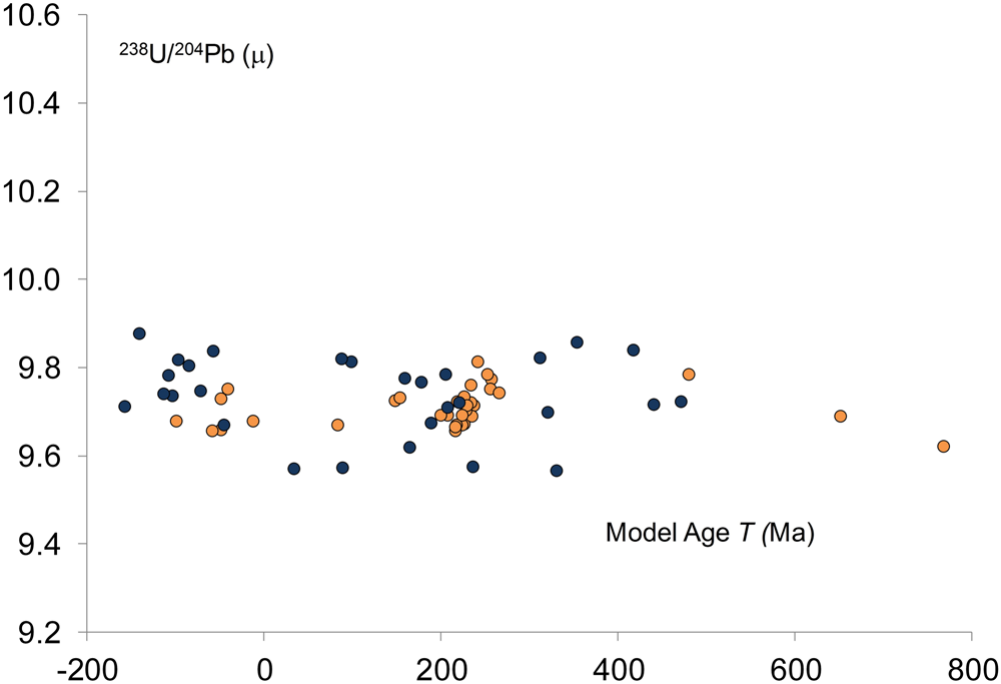
A comparison of the isotope composition of labile Pb in modern and ancient soils. The data from the modern soils was produced by leaching modern soil samples with deionised water. This modern data () is compared with the Pb isotope composition of bone and dentine from archaeological sites. The assumption made is that the bone and dentine re-equilibrated with the labile soil component close to the time of burial and thus provide a measure of labile Pb that predates modern pollutants ().

Bio-available Pb from modern British soils defines a broadly horizontal field of data with ^238^U/^204^Pb = 9.74 ± 0.18 (2 SD, n = 29) Fig. 1. The bio-available Pb from ancient soils, as represented by archaeological bone and dentine composition, give a comparable result of ^238^U/^204^Pb = 9.70 ± 0.08 (2 SD, n = 34). Both these results are in agreement with the average crust composition of ^238^U/^204^Pb = 9.7^22^ and synonymous with recycled sedimentary rocks. Some samples give negative model ages which is common in samples from limestone terrains and caused by a disproportionate uptake of U compared to Pb in marine carbonates^23^.

**Figure 2:**
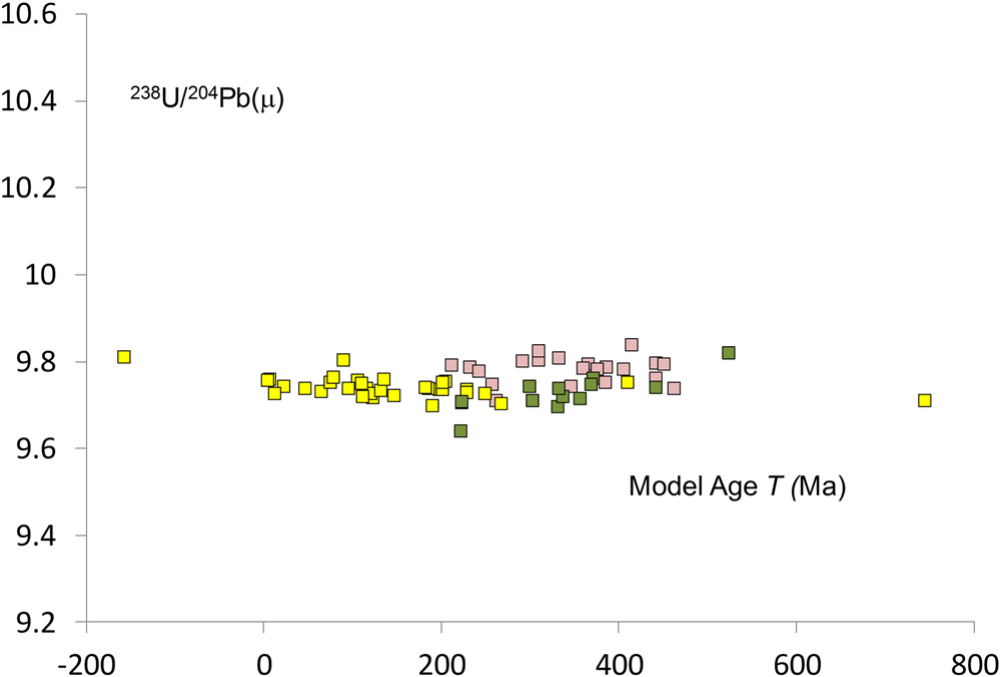
Natural Pb exposure. A ^238^U/^204^Pb (μ) vs *T*_(model age)_ for Late Neolithic pigs () and humans () and post Neolithic low Pb exposure individuals ().

The transfer of the bio-available soil Pb into fauna is shown in Fig. 2. Data from the Neolithic pigs tooth enamel range between model ages (*T)* of 209 and 471 Ma with ^238^U/^204^Pb = 9.78 ± 0.05 (2 SD, n = 23). Human tooth enamel data yields a similar ^238^U/^204^Pb = 9.73 ± 0.06 (2 SD, n = 56). The coincidence of the soil and faunal data fields provides firm evidence that natural Pb exposure is consistent with the ingestion of the bioavailable component of Pb in silicate based soil.

**Figure 3:**
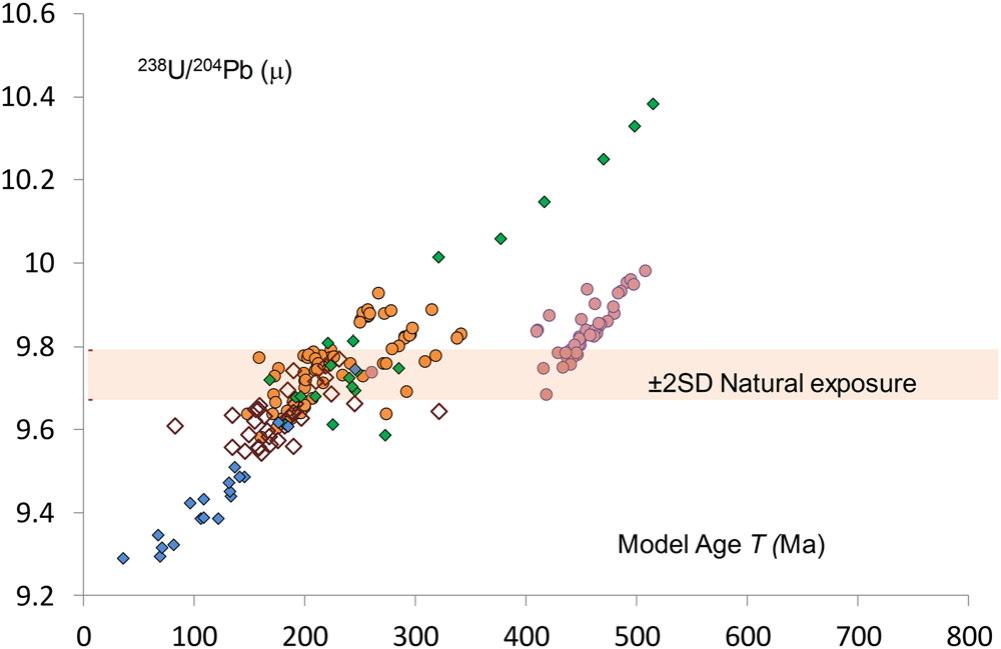
^238^U/^204^Pb (μ) vs *T*_(model age)_ for Anthropogenic Pb exposure. Anglo Saxon and Anglian human tooth enamel from Berinsfield (), Eastbourne () and West Heslerton () data fields compared with English () and Welsh () Pb isotope data from Galena. The extent of 238U/204Pb (μ) attributable to natural exposure is given as the 2SD range derived from the data used in Fig. 1.

Figure 3 shows the Pb isotope composition of tooth enamel from 5–7^th^ century individuals whose elevated Pb concentrations is taken as evidence of anthropogenic Pb exposure. The figure includes data from galena (PbS) in British deposits of the Mendips, Pennines and central Wales for comparison. The most obvious aspect of the diagram is the steeply sloping data fields created by both the tooth data and the galena compositions. The central Wales data best highlights the highly correlated nature of the ore composition, created during the process of mineralization. Similar arrays can be seen in many galena datasets^14^. The tooth enamel samples from West Heslerton and some of the Eastbourne samples plot close to those of the English galena compositions suggesting the Pb exposure of these individuals was dominated by British ore. However, six of the Eastbourne samples, and most of the Berinsfield data, extend beyond the range of the British deposits suggesting that some individuals carry a component of non-British Pb.

The Pb is locked into tooth enamel during mineralization, which for the M2 teeth of this study, occurs between two and eight years age^24^. There are a number of options for the Pb source and ingestion route. The main route for modern children’s Pb exposure is though hand to mouth soil ingestion^25^. However non-local Pb isotope signatures can arise from a number of routes: (1) The individual was exposed to Pb somewhere other than where they were found ie they are not of local origin (2) They were exposed to Pb from a non-local source^1^, and (3) They inherited a non-local Pb composition from their mother via placental^26^ or lacational^27^ transfer that was available or re-mobilized during tooth mineralization.

Strontium and oxygen isotope analysis has also been undertaken on these samples but does not support a non-British childhood for the majority of individuals from the Berinsfield and Eastbourne sites^17,28^ and so we rule out an immigrant population.

Thus the most likely exposure routes would appear to be either from imported goods or, that these are first generation arrivals whose mothers carried and transferred a Pb isotope signature from her homeland^29^; or it may be a combination of both. Grave goods from Berinsfield highlight continental connections^17^.

Some constraints can be placed on the geological origin of the Pb these people were exposed to: the Berinsfield array indicates an end-member of a geologically young, low-U Pb terrain, whereas the Eastbourne upper end-member indicates a U-rich terrain that is *c*. 600 Ma old. Lead and Pb-bearing silver deposits with isotope compositions similar to those seen in the Eastbourne and Berinsfield populations can be found in Europe^14^ and hence this signature could have been introduced to England either by early Anglo-Saxon groups arriving in England or through trade and exchange of coins, ornaments or weaponry with continental populations.

This study shows that naturally derived, bio-available Pb from ingested soil is characterized by a horizontal data array in ^238^U/^204^Pb-*T* space, which is mimicked by fauna exposed to this type of Pb. In contrast, sulphide ore deposits define steeply dipping data arrays, a trend that is also reflected in the tooth enamel of people who have been exposed to anthropogenic Pb. This difference in the orientations of the fields can thus be used to distinguish between natural and anthropogenic exposure.

It is proposed that this approach to characterizing the origin of human Pb exposure provides an alternative method to examining Pb sources, regardless of exposure levels, and allows new insights into the rise of mining during the Anthropocene^30^, development of metal working and trade in the ancient world and its impact on human health.

## Electronic supplementary material

Supplementary Information
