# Supplementary material for: Tracking natural and anthropogenic Pb exposure to its geological source
Source: Sci Rep. 2018 Jan 31;8:1969. doi: 10.1038/s41598-018-20397-y (PMC5792464; doi:10.1038/s41598-018-20397-y)
Supplement: Supplementary file 1 — Supplementary Information [file 41598_2018_20397_MOESM1_ESM.pdf]

Tracking natural and anthropogenic Pb exposure to its geological source

Jane Evans[1], Vanessa Pashley[1], Richard Madgwick[2], Samantha Neil[3], and Carolyn Chenery[1].

1) NERC Isotope Geosciences Laboratory, British Geological Survey, Keyworth, Nottingham, UK, NG12 5GG

2) Department of Archaeology, Cardiff University, Cardiff, Wales, CF10 3AT, UK

3) Department of Archaeology, Durham University, Durham, England, DH1 3LE

Supplementary Information

The data in this paper has been acquired over a number of years and includes Pb isotope data produced by both TIMS (Thermal Ionisation Mass Spectrometry) and MC-ICP-MS (Multi Collector Inductively Coupled Plasma Mass Spectrometry). Analytical methods, applicable to all data published here for the first time, are given below. Details of the methods for previously published data can be found in the relevant papers.

Source of samples and permissions:

Archaeological material used in this study comes from a number of sources. Some of the data are already published and some are new to this study. All are documented in the data table below.

Sample prefix	Site	Age	Source of Pb analysis	Permission for Pb analysis
AB	Boscombe/Wiltshire	Bronze Age	This study	Alastair Barclay
WHIT	Whitwell/Yorkshire	Neolithic	This study	Andrew Chamberlain
BER	Berinsfield/Oxfordshire	Anglo Saxon	This study	NIGL archive data
EAS	Eastbourne/East Sussex	Anglo Saxon	This study	NIGL archive data
TY	Ty Isaf, Monmouth	Neolithic	This study	National Museum Wales
MH	Mine Howe, Orkney	Scottish Iron Age	Montgomery et al [1]	
GRIS	Gristhorpe, Yorkshire	Bronze Age	Montgomery et al [1]	
S0025	Sedgeford, Norfolk	Anglo Saxon	This study	Lorraine Horsley
WASP	Wasperton/Warwickshire	Anglo Saxon	This study	Martin Carver
GEMAS	British GEMAS samples	Britain	This study	Clement Reimann
BIP	Britain	Bronze Age	This study	Mike Parker Pearson
WEY	Weymouth	Viking Age	Loe et al[2], Chenery et al[3].	
DW	Durrington Walls, Wiltshire	Neolithic	This study	R. Madgwick?
HR/BH/EC	Eastbourne/East Sussex	Anglo Saxon	This study	Jo Seaman
All other samples			Montgomery 2002 [4].	

The modern soils samples used in this study are from the UK GEMAS (Geochemical mapping of agricultural and grazing land soil) collection. Reimann et al[5] provide full details of the GEMAS data, including sample preparation; The locations for samples used here are presented in the table below.

Sample	Location	Longitude	Latitude
GEMAS 005	Shetland	-0.8785	60.7997
GEMAS 033	Aberdeenshire	-2.5760	57.3629
GEMAS 118	Dunbartonshire	-4.0524	55.5242
GEMAS 062	Buckinghamshire	-0.4763	51.5861

GEMAS 063	Wiltshire	-1.5958	51.4614
GEMAS 044	Devon	-4.4503	50.4722
GEMAS 012	Sutherland	-5.2422	58.1551
GEMAS 057	Hampshire	-0.2194	50.9625
GEMAS 006	Orkney	-3.2126	59.0674
GEMAS 003	Shetland	-1.3033	59.8868
GEMAS 014	Isle of Lewis	-6.5448	58.1649
GEMAS 074	Northamptonshire	-0.9433	52.4133
GEMAS 108	County Antrim	-6.7221	54.8095
GEMAS 040	Loch Aber	-4.7266	56.885
GEMAS 072	Cambridgeshire	0.6792	52.3192

Ancient soil Pb isotope compositions were determined from dentine which, like bone, re-equilibrates with its environment during burial[6 7], thus providing a bioavailable soil Pb isotope composition at the time of burial,

Sample preparation:

Soils: A gram of soil was shaken with de-ionized water and left over night. The samples were centrifuged and the supernatant fluid removed and transferred to a Savillex beaker. 8MHNO_3 was added to remove organic material and the sample was dried down and taken up in 0.5MHBr.

Enamel: Enamel samples were abraded to a depth of >100 microns, using a tungsten carbide dental burr, and the removed material discarded. A sample was then cut from the tooth using a flexible diamond edged rotary dental saw. All surfaces of the sample were mechanically cleaned with a tungsten carbide burr used to remove adhering dentine. The resulting enamel samples were transferred to a clean (class 100) laboratory suite where they were , leached for 5 minutes in a mixture of dilute HCl and HNO_3 acid, to remove any surface contamination, then rinsed several times with high purity water before being dried and dried and finally placed into pre-cleaned Teflon beakers.

Dentine: Where a dentine sample was needed the drilled powder from within the tooth was collected and set aside. Aliquots of the dentine powder was also placed in pre-cleaned Teflon beakers.

The enamel and dentine samples were then dissolved in Teflon distilled 8M $HNO_{3,}$ dried down, and taken up in 0.5MHBr.and lead was collected from all the samples using conventional anion exchange (Dowex AG-1) methods[8].

Isotope analysis:

TIMS: samples BER and EAS.

The Pb isotope composition and concentrations were determined by Thermal Ionisation Mass spectrometry (TIMS) using a Finnigan Mat 262 multi-collector mass spectrometer at NIGL. Pb was run using rhenium filaments in silica gel -phosphoric acid. Pb blanks are c 70pg. Lead isotope ratios were normalised to Todt et al values for the reference solution NBS 981 [9].Reproducibility, during the period of analysis, was: $^{206}Pb/^{204}Pb$= 0.20 % , $^{207}Pb/^{204}Pb$=0.29%, $^{208}Pb/^{204}Pb$=0.40%, (2SD, n=31). These data are from 20ng loads of the standard.

MC-ICP-MS: samples GEMAS, BIP, HR2, EC, BH, WASP.

Lead isotope analysis of these samples was conducted using a Nu Instruments Nu Plasma, MC-ICP-MS. Prior to analysis, each sample was spiked with a thallium (Tl) solution, which was added to allow for the correction of instrument induced mass bias. Samples were then introduced into the instrument via an ESI 50µl/min PFA micro-concentric nebuliser attached to a de-solvating unit, (Nu Instruments DSN 100). Faraday collectors were configured to allow the simultaneous detection of the following ion beams: 204Pb, 206Pb, 207Pb, 208Pb. Additionally: 203Tl and 205Tl for mass bias correction, and 202Hg to allow correction of the 204Hg interferant on 204Pb. Each individual

acquisition consisted of 60 ratios, collected at 5-second integrations, following a 60 second de-focused baseline.

The precision and accuracy of the method was assessed through repeat analysis of an NBS 981 Pb reference solution, (also spiked with Tl). The average values obtained for each of the mass bias corrected NBS 981 ratios were normalised to the known values for this reference, (Pb double spike data taken fromThirlwall, 2002: $^{206}Pb/^{204}P$ = 16.9417, $^{207}Pb/^{204}Pb$ = 15.4996, $^{208}Pb/^{204}Pb$ = 36.724, $^{207}Pb/^{206}Pb$ = 0.91488, $^{208}Pb/^{206}Pb$ = 2.1677 The analytical errors, reported for each of the sample ratios, are propagated relative to the reproducibility of the NBS 981 for each session, to take into account the errors associated with the normalisation process. Average 2SD reproducibility is: $^{206}Pb/^{204}Pb$ =0.008%; $^{207}Pb/^{204}Pb$ =0.008%; $^{208}Pb/^{204}Pb$ =0.009%.

Sample: Neolithic Pigs (DW): Due to the exceedingly low levels of Pb in these samples the Pb isotope composition of the pig enamel was determined using a Thermo Fisher Neptune Plus MC-ICP-MS equipped with a high sensitivity Jet interface. Samples were introduced into the instrument via an ESI 50µl/min PFA micro-concentric nebuliser attached to a de-solvating unit, (Cetac, Aridus II). All isotopes of interest were again detected simultaneously. Data handling procedures are the same as those detailed above. The external reproducibility, after propagation of standards uncertainty, is: $^{206}Pb/^{204}Pb$ =0.027%; $^{207}Pb/^{204}Pb$ =0.031%; $^{208}Pb/^{204}Pb$ =0.041%. The normalised and error propagated sample data is presented in Table 1. Procedural blanks were c 50pg.

Pb concentrations: samples HR2, EC, BH.

Pb concentrations were determined as follows: enamel samples were weighed into Ependorph centrifuge tubes and first cleaned by submersion for one minute in a mixed solution of 1%HNO_3 and 0.5%HCl. This was followed by rinsing (x3) in MilliQ de-ionized water. The samples were then dried. The samples were dissolved in 0.2ml of a 2:1 mixture of 8MHNO_3 and 6MHCl. Once the enamel was dissolved the solution was made up to 10mls using de-ionised water. The enamel Pb concentrations were determined at the British Geological Survey, Keyworth, UK, using an Agilent 7500cx quadrupole ICP-MS. The instrument was calibrated using a series of synthetic chemical solutions diluted from multi-element stock solutions (SPEX Certprep), the calibration being validated using synthetic chemical standards from a separate source. Digest solutions were diluted such that the calcium concentration was between 100 and 200 ppm, optimal for long-term instrument stability, detection, and calibration ranges. The reproducibility of the Pb concentration data is ±10% (2SD).

Geological comparison of silicate and sulphide Pb isotope systems.

Approximately 90% on the earth's crust is composed of silicate minerals and, of these, the most common are quartz (SiO2) and feldspars ($KAlSi_3O_8$ – $NaAlSi_3O_8$ – $CaAl_2Si_2O_8$). These minerals originate though the crystallisation of magma, either above or below ground, to create igneous rocks, such as granite. At the point of cooling the isotope systematics of these minerals are locked in and the radiometric clock starts ticking. Such rock formations can be eroded and redeposited as sedimentary rocks, but the minerals are robust and retain their isotope integrity[10] so that, through constant reworking and re-deposition by geological processes, the crust achieves a typical "average" isotope composition. However, when events, such as mountain building, occur the crust is subjected to high temperatures and pressures. Hydrothermal fluids infiltrate and leach elements from the crustal rocks. As the waters cool, the new minerals crystallise out and deposit their elemental load in rich

concentrations of metal bearing minerals, commonly in the form of sulphides such as: Galena (PbS), Sphalerite (ZnS), Chalcopyrite ($CuFeS_2$), Stibnite (Sb_2S_3), and Pyrite (FeS_2). The process of hydrothermal alteration causes re-homogenization (resetting) of the isotope systems and establishes a relationship between the geochemical composition of the system and its new starting isotope composition, which is reflected in correlated arrays of data. The effect is well described for both mineral[8] and whole rocks [11] in the Rb-Sr decay scheme.

Site and sample descriptions pertaining to data published here for the first time[12]:

Neolithic: Whitwell, Derbyshire [13]

Whitwell Cairn is located approximately 20 miles (32 kilometres) to the north of the city of Nottingham in the county of Derbyshire, England[13]. Whitwell cairn was discovered in 1988 during excavation of Whitwell Quarry, between the villages of Creswell and Whitwell in Derbyshire, SK 53207482. The cairn was constructed just beneath the summit of a north-south orientated limestone ridge. To the east the land dips more gradually, the limestone eventually running under the Bunter Sandstone beds. It is estimated that the first individuals to be buried at Whitwell died during the 38th century BC.[13,14]

Neolithic: Ty Isaf, Monmouth, Wales [15]

Ty Isaf is a long cairn, located within a cluster of monuments in the Black Mountains, Powys, and south-eastern Wales. Monuments in the Black Mountains form part of the Cotswold-Severn group, a concentration of long cairns found in the regions that surround the Severn Estuary: south-east Wales, the Cotswolds, Somerset and Wiltshire in southern Britain [16]. Ty Isaf is a composite monument consisting of two distinct elements: a long cairn and a circular rotunda [17]. Individuals buried within Ty Isaf have been radiocarbon dated to the mid 4th millennium to earlier 3rd millennium BC. [12, 14.] Tooth enamel from four samples have been analysed for Pb isotopes in this study (39.190/201 LM2 and LM3, and 39.190/148bLM2 and LM3).

Late Neolithic: Durrington Walls, Wiltshire [18,19]

Durrington Walls is a large henge enclosure located c. 3km northeast of Stonehenge, Wiltshire, UK. It has produced substantial assemblages of animal bone and ceramics that are strongly suggestive of feasting. The peak period of activity at the site centres on 2500 B.C., when it formed the largest known settlement in northwest Europe, perhaps housing more than 4000 people seasonally. The Pb isotope composition of twenty-one samples of porcine enamel (all prefixed DWP) are presented in the data table.

Bronze Age samples.

Although the Bronze Age saw the onset of mining and metal work, the exposure to anthropogenic Pb in the overall population seems very restricted. The median Pb concentration of 13 British Bronze age individuals is 0.06 ppm [1]. Three Bronze Age samples are used in this study as part of the natural exposure data set: Gristhorpe man (Pb ppm = 0.003) and two teeth (M3 and PM2) from AB61231, which are from a Bronze Age individual from Amesbury in Wiltshire. Pb concentrations were not determined for the Amesbury sample but are assumed to be within the natural range of the British Bronze age given above.

Scottish Iron Age: Mine Howe

Two sample, MH05 1861 and MH 04 897 are included in the natural exposure dataset [1].

Anglo Saxon: Berinsfield, Oxforshire [20]

The Berinsfield Anglo-Saxon cemetery is located 1.1 km northeast of Dorchester-upon-Thames and 300 m northwest of the River Thame. Salvage excavations ahead of gravel extraction, undertaken by the Oxford Archaeological Unit in 1974 and 1975, uncovered 114 early Anglo-Saxon inhumations,.

The graves were placed within a system of Romano-British rectangular field enclosures bounded by north/south oriented ditches showing evidence of use throughout the Roman period (A.D. 59 -410). The cemetery is typical of those identified as early Anglo-Saxon[21] with irregularly-spaced and -oriented graves. Associated with the cemetery are typical Germanic grave furnishings, e.g. brooches, necklaces, toilet sets, belt buckles, and other objects of personal dress, as well as weaponry, knives, buckets, pottery [22]. These items, and several radiocarbon dates, date cemetery use from the early/mid-fifth century A.D. to the early 7th century A.D.[22] (Samples from this site are prefixed BER)

Anglo Saxon Eastbourne, Sussex [23]

The Eastbourne Anglo-Saxon cemetery is located in the town of Eastbourne on the south central coast of England. Grave goods, buried with many of the individuals here suggest that the Eastbourne Anglo-Saxon cemetery is represented by burials dating from the late 4th century to the 7th century A.D, of which a sample was selected dating between 375 and 600 AD.

Viking Age, Weymouth, Dorset.[3]

The Viking Age samples are from a mass execution pit, where at least 51 adult male individuals were discovered during the construction of a road at Ridgeway Hill, north of Weymouth, Dorset, in Southern England. All of the men had been decapitated, with the skulls and mandibles deposited in a pile at the southern edge of the pit, while the postcranial remains were deposited, apparently with little care, on top of one another across the rest of the pit [2]. All of the skulls were male, and many appeared to be young adults with a few older males. This interpretation is supported by evidence from the postcranial remains, the study of which classified 70% as under the age of 25[2]. Thirty-four skulls have evidence of sharp-force trauma and, in many cases, show multiple injuries. AMS radiocarbon dating of three individuals gave dates that are statistically consistent. Their weighted mean, when calibrated, provides a date range of AD 970–1025 (93% probability). Samples from this site are all prefixed with WEY. A further Viking age sample from Ardnamuchan is included in this group[24].

Appendix Figure 1. A conventional $^{206}Pb/^{204}Pb$ vs $^{208}Pb/^{204}Pb$ diagram displaying all the data presented in this study in Figures 1, 2 & 4 with uncertainty ranges given for TIMS and Plasma analysis.

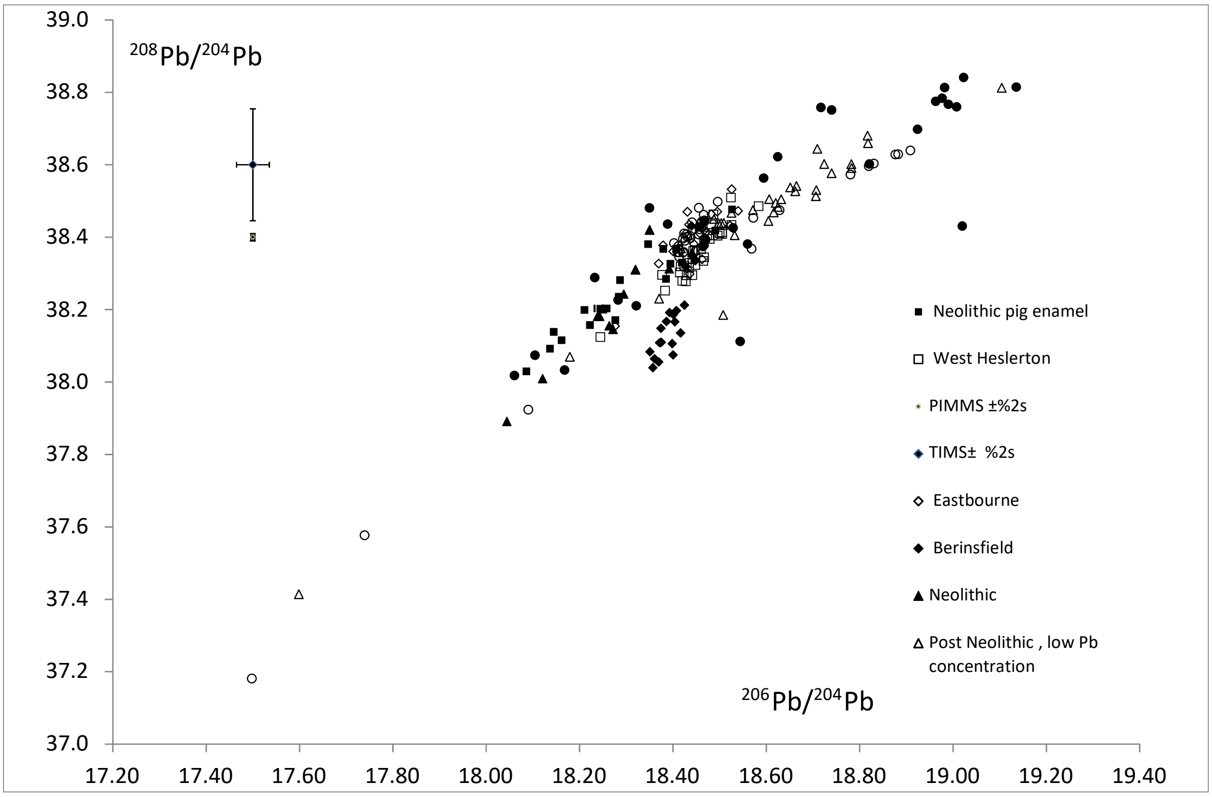

1 Montgomery, J., Evans, J. A., Chenery, S. R., Pashley, V. & Killgrove, K. in *Roman diasporas: archaeological approaches to mobility and diversity in the Roman Empire.* (ed H. Eckardt) 204-226 (Journal of Roman Archaeology Suppl. 78, 2010).

2 Loe, L., Boyle, A., Webb, H. & Score, D. *"Given to the ground" A Viking Age Mass Grave on Ridgway Hill, Weymouth.*, (Berforts Information Press, 2014).

3 Chenery, C., Evans, J. A., Score, D. & Boyle, A. A boat load of Vikings? *Journal of the North Atlantic* **Special Volume 7**, 43-53 (2014).

4 Montgomery, J. *Lead and strontium isotope compositions of human dental tissues as an indicator of ancient exposure and population dynamics* PhD thesis, University of Bradford, (2002).

5 Reimann, C., Birke, M., Demetriades, A., Filzmoser, P. & O'Conner, P. *Chemistry of Europes Agricultural soils. Part A: Methodology and Interpretation of the GEMAS dataset.*, Vol. B 102 (2014).

6 Trickett, M. A., Budd, P., Montgomery, J. & Evans, J. An assessment of solubility profiling as a decontamination procedure for the Sr-87/Sr-86 analysis of archaeological human skeletal tissue. *Appl. Geochem.* **18**, 653-658 (2003).

7 Dudás, F. Ö., LeBlanc, S. A., Carter, S. W. & Bowring, S. A. Pb and Sr concentrations and isotopic compositions in prehistoric North American teeth: A methodological study. *Chem. Geol.* **429**, 21-32 (2016).

8 Dickin, A. P. *Radiogenic Isotope Geology*. (Cambridge University Press, 1995).

9 Todt, W., Cliff, R. A., Hanser, A. & A.W.Hofmann. Re-calibration of NBS lead standards using a 202Pb+205Pb double spike. *Terra Abstracts* **5**, 396 (1993).

10 Zengjie Zhang *et al.* Pb isotope compositions of detrital K-feldspar grains in the upper-middle Yangtze River system: Implications for sediment provenance and drainage evolution. *Geochem. Geophys. Geosyst.* **15**, 2765-2779, doi:10.1002/2014GC005391 (2014).

11 Evans, J. A., Millar, I. L. & Noble, S. R. Hydration During Uplift Is Recorded by Reset Rb-Sr Whole-Rock Ages. *J Geol Soc* **152**, 209-212 (1995).

12 Neil, S. *The application of strontium and oxygen isotope analysis to study land use and mobility patterns during the earlier Neolithic in England and Wales.* PhD thesis, Durham, (2017).
13 Vyner, B. a. W., I. A Neolithic cairn at Whitwell, Derbyshire. *Derbyshire Archaeological Journal* **131**, 1-131. (2011).
14 Griffiths, S. *Chronological modelling of the Mesolithic–Neolithic transition in the midlands and north of England.* PhD thesis, Cardiff University., (2011).
15 Neil, S., Montgomery, J., Evans, J., Cook, G. T. & Scarre, C. Land use and mobility during the Neolithic in Wales explored using isotope analysis of tooth enamel. *American Journal of Physical Anthropology* (in press).
16 Darvill, T. *Long barrows of the Cotswolds and surrounding areas, Stroud.*, (Tempus., 2004).
17 Grimes, W. F. The excavation of Ty-Isaf long cairn, Brecknockshire. . *Proceedings of the Prehistoric Society* **5**, 119-142 (1939).
18 Madgwick, R. *et al.* Multi-isotope analysis demonstrates long distance movement of people and animals for feasts in the Stonehenge landscape. *Nature reports* (submitted).
19 Parker-Pearson, M. *Stonehenge: Exploring the greatest Stone Age mystery*. (Simon and Schuster, 2012).
20 Hughes, S. S. *et al.* Anglo-Saxon origins investigated by isotopic analysis of burials from Berinsfield, Oxfordshire, UK. *J. Arch Sci.* **42**, 81-92, doi:10.1016/j.jas.2013.10.025 (2014).
21 S., L. *The Anglo-Saxon way of death.* . (2000).
22 A, B., A, D. & D, M. *An Anglo-Saxon Cemetery at Berinsfield, Wally Corner. editors.* . Vol. 1-197 (Oxford Archaeological Unit., 1995).
23 Hughes, S. S., Millard, A. R., Chenery, C. A., Nowell, G. & Pearson, D. G. Isotopic Analysis of burials from the early Anglo Saxon cemetery at Eastbourne, Sussex, UK. (submitted).
24 Harris, O. J. *et al.* Assembling places and persons: a tenth century Viking boat burial from Swordle Bay, on the Ardnmurchan peninsula, western Scotland. *Antiquity* **91**, 191-206, doi.org/10.15184/aqy.2016.222 (2017).
